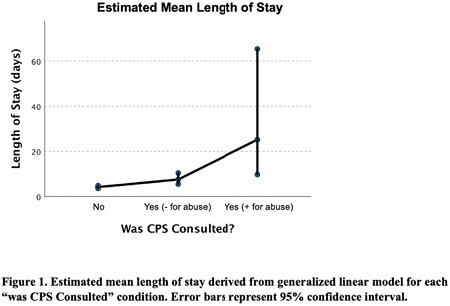# 96 The Influence of Consulting Child Protective Care Services on Patients’ Length of Stay in Burns

**DOI:** 10.1093/jbcr/irae036.095

**Published:** 2024-04-17

**Authors:** Marian Mikhael, Bilal Koussayer, Timothy Nehila, William West, III, Julia A Morris, Nicole K Le, Kristen Whalen, Kristina Buller, Jeegan Parikh, Luba Ayzenshtat, Jared Troy, Jake Laun

**Affiliations:** Morsani College of Medicine USF, Tampa, FL; Tampa General Hospital, Tampa, Florida; Morsani College of Medicine USF, Tampa, FL; Tampa General Hospital, Tampa, Florida; Morsani College of Medicine USF, Tampa, FL; Tampa General Hospital, Tampa, Florida; Morsani College of Medicine USF, Tampa, FL; Tampa General Hospital, Tampa, Florida; Morsani College of Medicine USF, Tampa, FL; Tampa General Hospital, Tampa, Florida; Morsani College of Medicine USF, Tampa, FL; Tampa General Hospital, Tampa, Florida; Morsani College of Medicine USF, Tampa, FL; Tampa General Hospital, Tampa, Florida; Morsani College of Medicine USF, Tampa, FL; Tampa General Hospital, Tampa, Florida; Morsani College of Medicine USF, Tampa, FL; Tampa General Hospital, Tampa, Florida; Morsani College of Medicine USF, Tampa, FL; Tampa General Hospital, Tampa, Florida; Morsani College of Medicine USF, Tampa, FL; Tampa General Hospital, Tampa, Florida; Morsani College of Medicine USF, Tampa, FL; Tampa General Hospital, Tampa, Florida

## Abstract

**Introduction:**

Child endangerment commonly involves abuse, including burns. Healthcare professionals may feel that consulting Child Protective Services (CPS) unnecessarily prolongs a patient’s length of stay (LOS), as most cases are determined to be not significant for abuse. This study was conducted to determine the relationship between CPS consultations and patients’ LOS.

**Methods:**

We conducted a retrospective cohort study of pediatric patients under the age of 18 years that were admitted at our tertiary healthcare institution between 2020-2021 for burns. We examined the LOS for patients in which CPS was not consulted (Control), CPS was consulted without evidence of child endangerment (-CPS), and CPS was consulted with evidence of child endangerment (+CPS). A secondary analysis was conducted only on patients who required inpatient burn care by removing patients that did not undergo surgical intervention.

**Results:**

Of the 158 patients admitted, 33 patients had CPS involved. There was no significant difference between LOS in the control and CPS consultation (- for abuse) patients (p=0.086). There was also no significant difference between LOS in the control and CPS consultation (+ for abuse) patients (p=0.150). These findings persisted controlling for age, total burned surface area (TBSA), and number of surgeries for burns.

In the secondary analysis, 104 patients did not undergo surgical intervention and were removed. The effect of consulting CPS on LOS in the remaining 54 patients did not change (p>0.05).

**Conclusions:**

This study demonstrates that CPS should be consulted when there is any suspicion of child endangerment without concern for unnecessarily increasing the patient’s LOS.

**Applicability of Research to Practice:**

CPS plays a vital role in burn consultations for pediatric patients by identifying risk factors for endangerment, ensuring the welfare of children, and providing legal protection. No previous studies have explicitly examined the relationship between CPS consultation and LOS in pediatric burn patients or in any other child abuse victims due to other etiology. In some instances, there may be a hesitancy to contact CPS when there is doubt about non-accidental trauma and concern for unnecessary hospital admission, however our study results suggest that CPS involvement does not affect the LOS of patients. Early identification and intervention can help protect these patients, and establishing standardized protocols for reporting suspected child abuse cases is crucial to ensure that at-risk patients receive the proper consultations in a timely manner.